# The Greek smoking epidemic from a life-course perspective

**DOI:** 10.1093/pubmed/fdab342

**Published:** 2021-09-08

**Authors:** Rebekka Christopoulou, Georgios Mavropoulos, Georgios Voucharas

**Affiliations:** Department of Economic Sciences, University of Macedonia, 156 Egnatia Str, Thessaloniki 54636, Greece; Department of Economic Sciences, University of Macedonia, 156 Egnatia Str, Thessaloniki 54636, Greece; Department of Economic Sciences, University of Macedonia, 156 Egnatia Str, Thessaloniki 54636, Greece

**Keywords:** cigarette epidemic, Greece, life course, smoking-attributable mortality, smoking prevalence

## Abstract

**Background:**

Smoking rates in Greece are the highest recorded among OECD countries, but the historical and life-course evolution of smoking patterns is largely unknown. The present paper addresses this gap.

**Methods:**

We produce nationally representative life-course trajectories of smoking and related mortality of eight generations of Greek men and women. We estimate the smoking–mortality correlation conditional on several confounders and project the estimates forward.

**Results:**

We show that smoking prevalence among Greek men has plateaued at >60% for all but the youngest generation. For women, smoking prevalence is relatively lower, lags by several generations and follows a hump-shaped pattern. Smoking-attributable mortality is currently peaking for men (nearing 40% of total deaths) and is rising for women. We estimate that it takes ~20 years of smoking to maximize the smoking–mortality correlation (at 0.48 for men and 0.32 for women). Based on this estimation, we forecast that mortality rates will begin falling within the current decade.

**Conclusions:**

The breadth of the Greek smoking epidemic has been high by international standards, reflecting the ineffective tobacco control efforts in the country. While smoking popularity fell during the Great Recession, policy vigilance is necessary to prevent a relapse once the economy recovers.

## Introduction

Despite the significant decline in cigarette consumption recorded in Greece during the late 2000s,^1^^,^^2^ smoking remains alarmingly popular. Based on the most recent available statistics in 2017, one out of three Greeks over the age of 15 smoked cigarettes daily—the highest percentage recorded among all OECD (Organisation for Economic Co-operation and Development) countries that year.^3^

Tackling smoking became more urgent than ever during the recent ‘Great Recession’. Smoking entails a range of economic burdens on the Greek society, including its direct medical costs, which weigh on the already drained public health resources. Tsalapati *et al*.^4^ estimate that over €554 million was spent in 2011 on hospital costs for treatments of smoking-attributable conditions, a sum that represents 10.7% of the national hospital budget. Adding to the equation other types of smoking-related costs raises this sum to €3.4 billion annually, representing ~15% of the total health expenditure.^5^ More recently, the outbreak of COVID-19 has further exacerbated the health costs of smoking due to its association with the negative progression and the adverse outcome of the disease.^6^^,^^7^

Given its vigilance on tobacco control measures, the current government seems to understand the severity of the situation.^8^ However, the information needed to effectively design and target policies is to a large extent missing. Due to data limitations, existing smoking research in Greece relies either on cross-sectional measures of individual smoking behavior^1^^,^^9^^,^^10^ or on short time-series of aggregate measures of cigarette consumption.^11^ This type of research has yielded snapshots of smoking patterns and their determinants, aggregate estimates of demand elasticity for cigarettes and assessments of anti-smoking interventions. While the policy relevance of this output is unquestionable, it only goes some distance. Two important gaps remain.

First, the historical evolution of smoking in the country is largely unknown. Apart from the time-series of cigarette consumption, which starts in 1960 and is not measured consistently to date, there is no other indicator of the country’s smoking history. Cross-sectional studies provide sporadic estimates of the smoking prevalence, but most are recent and/or based on unrepresentative samples. Because researchers and policymakers do not know how the popularity and intensity of smoking spread and which generations were affected the most, they cannot precisely predict the prevalence of smoking-related diseases in the future and its distribution across the population. Second, smoking behavior does not only differ across individuals but also over the life course of each individual and, presumably, so does its responsiveness to tobacco control policies. It is this life-course dimension that the existing literature is lacking.

This paper aims to fill both gaps. Using retrospectively reported data that describe smoking behavior over each person’s life, we reconstruct gender- and generation-specific smoking trajectories, and we relate these to the corresponding trajectories of smoking-attributable mortality. Thus, for the first time, we fully describe smoking diffusion in Greece from the 1930s and we discuss our results in the context of the smoking epidemic model.^12^^,^^13^ Our paper joins an established literature that studies the dynamics of cohort-specific smoking behavior to inform policymaking.^14–33^

## Methods

The only population-wide survey in Greece that has collected retrospective smoking information is the Global Adult Tobacco Survey (GATS) which was administered in 2013. So far, Greek scholars have focused on the cross-sectional dimension of the data^9^ and have not taken advantage of the retrospective reports. Among others, the GATS asks whether a person currently smokes (including cigarettes, cigars and pipes), ever smoked in the past and when a person started and (if relevant) quit smoking.

Our calculations assume that a person smoked in each year from the age s/he reported starting until either the age s/he quit (ex-smokers) or the year of the survey (current smokers). Thus, we construct a smoking-status indicator for every person-year observation, which equals 1 if that person smokes and 0 if s/he does not. We then identify members of the same sex who were of the ages 10–19, 20–29,…, 80–89 in 2013, i.e. born in 1994–2003, 1984–93,…, 1924–33, respectively. We generate life-course smoking prevalence as the mean smoking status in each year by gender and birth-cohort (weighted by sampling weights).

A common concern regarding retrospective smoking data is that they can be limited by recall errors and other inaccuracies, but evidence suggests that this bias is small.^34–37^ More concerning is the fact that smokers die sooner than non-smokers, which causes retrospective smoking data to underestimate the prevalence rates for older cohorts. However, this too is manageable. The differential mortality bias affects the smoking trajectories of individuals who are older than 70 at the time of survey and can be addressed with a relatively straightforward correcting technique,^38^^,^^39^ which we apply here (see the online supplement).

As a last step, we construct cohort and gender-specific trajectories of smoking-attributable mortality by applying an established procedure by Peto *et al*.,^40^ which requires only widely available vital statistics (cause-specific deaths by gender, year and age-group from the WHO mortality database) and whose validity has been confirmed against other methods.^41^^,^^42^ We then correlate the cohort-specific, smoking-related mortality with the corresponding smoking prevalence 10–30 years earlier, controlling for several confounders. Specifically, for each lag distance *l* between smoking and mortality rates, we estimate different specifications of the following model:

$$ {M}_{c,t+1}=\alpha +\beta{S}_{ct}+\sum \limits_k{\gamma}_k{X}_{kt}+{e}_{ct}, $$

where *M* denotes smoking-attributable mortality rates; *S* denotes smoking rates; *X_k_* denotes a set of *k* control variables; *α* is the intercept; *β* is the correlation of interest; *γ_k_* denotes the effect of the *k*th control variable and *e* is the error term. Index *c* stands for birth-cohort, *t* stands for year and *l* takes values from 10 to 30 (i.e. for each specification, we run an overall of 21 regressions). This analysis allows us to identify the duration of cigarette consumption that is associated with the highest probability of premature death (i.e. the *l* that corresponds to the maximum estimated *β*) and to project smoking-attributable mortality forward (additional details are given in the online supplement).

## Results

### The smoking epidemic

To inform policy discussions on tobacco control, researchers commonly rely on the ‘cigarette epidemic’ model.^12^^,^^13^ The main idea in that model is that cigarette consumption follows a hump-shaped pattern while a country develops. Smoking uptake initially increases as economic development facilitates access to cigarettes, but it starts declining when deterrent factors co-evolving with development begin to dominate (e.g. educational attainment increases, health information spreads and anti-smoking measures are enforced). Importantly, this pattern in overall smoking rates translates into a similar pattern in smoking-attributable mortality a few decades later. Thun *et al*.^13^ specify four stages across which this co-movement occurs. At low levels of economic development, smoking diffusion is limited and smoking-related mortality is almost nonexistent (Stage I). As development increases, at first, smoking rates start spiraling while related mortality remains low (Stage II) but, over time, smoking loses popularity and mortality peaks (Stage III) before both fall to lower levels (Stage IV). The timing and severity of these trends differ by gender, with the epidemic affecting women later and less heavily when compared to men.

**Fig. 1:**
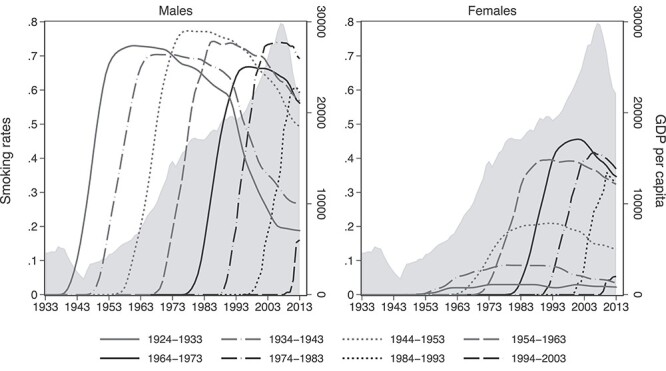
Smoking prevalence trajectories by gender and birth-cohort. Notes: the lines represent cohort-specific smoking trajectories; the shaded area plots GDP per capita.

Here, for the first time, we analyze Greek smoking patterns within the framework of the cigarette epidemic model. We start with Fig. 1 that plots the life-course evolution of smoking rates by generation and sex along with gross domestic product (GDP) per capita. When reading this figure, note that as each generation grows older, smoking rates increase to a peak and then fall, reflecting the dynamics in smoking initiation and cessation. Further, in generations whose smoking trajectory has higher kurtosis, people smoked fewer years. Finally, although the correspondence is not exact, a generation’s peak smoking prevalence approximates its share of ever-smokers. Thus, comparing peaks gives a picture of smoking diffusion across successive generations.

Several patterns are immediately apparent. First, as Greece grew consistently from the end of World War II till the recent Great Recession, the smoking rates of men remained persistently high (67–77%) with sporadic ups and downs. So, smoking diffusion among men forms a ‘bumpy’ plateau rather than a hump-shaped pattern as the economy expands. Smoking popularity among men fell slightly (at only 61%) for the generation that was in early adulthood during the Great recession and is much smaller for the generation that reached puberty during the same period, although this is not a safe conclusion since the data stop in 2013.

In all other aspects, the cigarette epidemic model is confirmed. Relative to men, smoking diffusion among women stalled by several decades and was more limited. Women first started smoking in large proportions in the 1960s and 1970s, as the second wave of feminism was unfolding and as they became the targets of cigarette advertising and branding.^43^ Smoking popularity among women initially rose across generations, peaked for those born during 1964–73 and ultimately fell among subsequent cohorts, though smoking rates in the youngest cohort continue to surpass those of the oldest cohort.

The similarities and differences in smoking behavior between genders are more apparent in Table 1. Relative to the older generations, women in recent generations have been starting to smoke at younger ages, whereas men still initiate smoking around the same age. By contrast, men and women in all birth-cohorts quit smoking at similar ages. Thus, women have been catching up with men in their age of initiation, in their age at peak smoking prevalence and in overall smoking duration. Encouragingly, gender parity in the duration of the smoking habit is achieved because years of smoking decrease across generations for both genders.

**Table 1:** Summary statistics of smoking patterns and related mortality by generation and gender

Sex	Birth-cohort	Observations	Peak smoking prevalence	Mean start age	Mean quit age	Age at peak smoking prevalence	Peak smoking-attributable mortality	Mean cigarettes per day (current smokers only)
		(1)	(2)	(3)	(4)	(5)	(6)	(7)
Males								
	1994–2003	83	0.16	17	18	17	0.05^a^	15
	1984–93	203	0.61	17	25	23	0.25^a^	19
	1974–83	361	0.74	18	32	28	0.38^a^	20
	1964–73	346	0.67	18	39	28	0.32^a^	22
	1954–63	255	0.74	18	43	27	0.37^a^	24
	1944–53	283	0.77	18	53	29	0.38	24
	1934–43	253	0.70	19	56	33	0.35	23
	1924–33	102	0.73	17	60	30	0.20	19
Females								
	1994–2003	71	0.05	16	18	17	0.09^a^	9
	1984–93	207	0.36	18	21	21	0.21^a^	18
	1974–83	401	0.42	19	30	27	0.23^a^	16
	1964–73	349	0.46	20	37	32	0.21^a^	17
	1954–63	289	0.40	22	42	33	0.18^a^	18
	1944–53	302	0.21	25	51	43	0.11	17
	1934–43	307	0.09	24	58	40	0.07	9
	1924–33	116	0.03	39	69	44	0.03	10

^a^Forecasted.

**Fig. 2:**
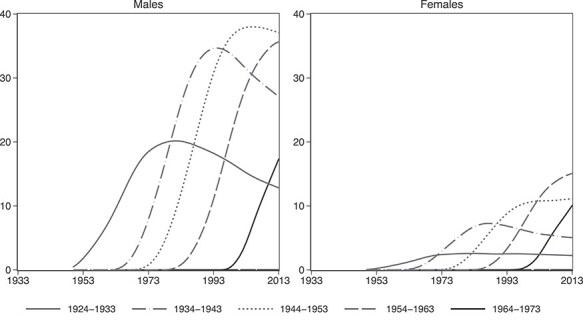
Trajectories of smoking-attributable deaths (as a % of total deaths) by gender and birth-cohort.

Figure 2 shows that these trends in smoking behavior across genders and generations translate into corresponding differentials in smoking-attributable mortality, which one misses when one pools genders and cohorts together. Like smoking rates, smoking-attributable mortality in each cohort increases to a peak and then falls, but that pattern occurs a few decades later. In our sample, individuals born after 1973 are younger than 40 years in 2013, which means that the life cost of smoking has not emerged yet for that group and is virtually zero. Thus, Fig. 2 contains the five oldest out of the eight generations in the sample. In combination with Fig. 1, Fig. 2 shows that the Greek smoking epidemic has been slow to mature and has only recently entered Phase III since smoking prevalence has started falling but mortality is currently spiraling to its peak (nearing 40% of total deaths). This agrees with the evidence that smoking prevalence among Greek physicians in the mid-2000s was comparable to that of the general population,^44^ which also indicates an immature epidemic.^45^

### The smoking–mortality correspondence

**Fig. 3:**
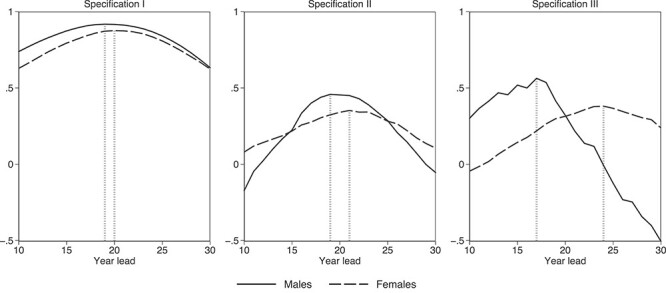
Estimated coefficients from regressions of smoking-attributable mortality on smoking prevalence at different temporal distances. Notes: Each point of each line represents a coefficient from a different regression. Observations are at the year-cohort level. Specification I includes no controls; Specification II controls for age and age-squared as well as for GDP (from the Maddison Project Database), health services as a share of GDP and physicians per capita (from the Hellenic Statistical Authority) and population (from the WHO mortality database), all observed at *t* and *t* + lead; Specification III controls for birth-cohort, age and year fixed effects. Vertical lines flag maximum estimated coefficients for each gender.

The natural question to ask next is how strongly and how fast smoking affects mortality. In Fig. 3, we plot estimates of the smoking-mortality correlation from three regression specifications. Specification I includes no controls and, therefore, estimates unconditional correlations. In Specification II, we control for health services as a share of GDP and physicians per capita to capture the changes in the supply of health care, population size to capture changes in health care demand, GDP per capita to capture dynamics in the overall quality of life and age and age-squared to net out relevant unobserved age-varying factors. In Specification III, we follow an even more conservative approach by including a full set of birth-cohort, age and year fixed-effects and, thus, we potentially over-control for unobserved variation.

In all cases, as the temporal distance grows between the years, mortality and smoking are measured, and their estimated correlation increases to a maximum value after which it gradually falls. Importantly, correlations reach their maximum value faster for men than for women, suggesting that smoking-related mortality for women smokers takes longer to unfold. This finding agrees with similar evidence from US data^28^ and arguably relates to the fact that women’s lung cancer survival rates are higher than men’s.^46^^,^^47^

Plausibly, inclusion of controls causes the smoking-mortality correlations to decrease. While Specification I produces a near one-to-one correlation, the corresponding estimate in Specifications II and III is less than half that size. In Specification II, which is the middle-ground model, we find that a 1 percentage point increase in the fraction of men who smoke is associated with an increase in smoking-attributable death rates that peaks at 0.48 percentage points 19 years later. The corresponding peak for women is at 0.32 percentage points 21 years later.

Using these peak correlation estimates from Specification II, we proceed to predict future smoking-attributable mortality. Under some alterable assumptions, we predict mortality rates both during the period in which smoking data are available and for two future decades; in this case, until the early 2030s. Focusing on the six cohorts that may survive until that time, we show that their predicted mortality trajectories replicate the observed trajectories fairly accurately (see Supplementary Fig. S2 for a color version). In column 6 of Table 1, we record the forecasted peak smoking-related death rates for the five youngest generations along with the observed peak rates for the three oldest generations. As expected, peak mortality and smoking rates increase and fall in sync across generations. An interesting finding is that mortality rates of women will exceed those of men starting with the youngest generation—though, this is indefinite since only the early part of the mortality trajectory can be forecasted for that generation. A more definite finding is that, for both genders, mortality rates are projected to begin falling within the current decade—a trend led by the two youngest generations.

## Discussion

### Main finding of this study

To summarize, we find that the popularity of smoking among men was persistently high for six generations, entailing an equally persistent mortality cost that is yet to fully unfold. By comparison, smoking among women diffused narrowly a few decades later, resulting in lower mortality cost that has only recently started to emerge. Based on these patterns, we infer that the Greek smoking epidemic is currently at Phase III; we estimate that the death risk for Greek smokers is maximized after 20 years of continuous smoking and we predict that smoking-related mortality will begin falling in the current decade.

### What is already known on this topic

Existing evidence on smoking in Greece is based on cross-sectional data or aggregate time-series.

### What this study adds

Our paper is the first to narrate the Greek smoking history using intergenerational life-course analysis. Compared with analogous evidence from 10 other countries, Greece ranks very high in terms of smoking prevalence for both men and women.^27^ Greece fares worse even when contrasted to Spain, a country with similar economic dynamics. Relative to the Spanish experience, smoking spread to a higher share of Greek men and a similar share of Greek women.

**Fig. 4:**
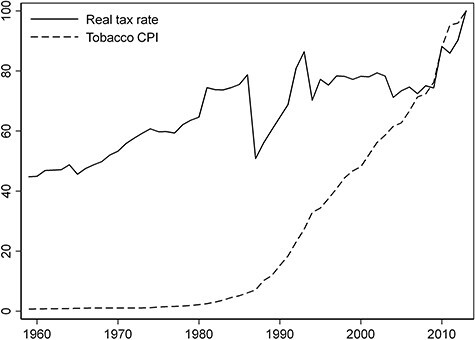
Real cigarette tax rate and tobacco consumer price index (CPI), 2013 = 100. Notes: The tobacco CPI is from the Hellenic Statistical Authority (ELSTAT). We set real tobacco taxes = tobacco tax revenue/cigarette consumption/tobacco CPI. Tobacco tax revenue measures excise duty in million Euro and is taken from the ELSTAT Yearbooks and OECD Statistics. Consumption of cigarettes counts millions of manufactured cigarettes and is from the International Smoking Statistics database, WEB Edition.

These patterns mirror Greek tobacco control efforts which have been problematic throughout the 20th century. Despite early health warnings from the scientific community, and aside from inconsistent baby-steps in cigarette tax policy (see Fig. 4) which had demonstratively low effectiveness,^48^^,^^49^ the first serious anti-smoking effort in Greece occurred in 1978. That effort involved the prohibition of tobacco advertising on broadcasting media and of smoking in enclosed public spaces. It also involved a wide information campaign, including broadcasts of anti-smoking messages on TV and radio and distribution of posters and booklets in schools and health care centers. While after the campaign, the annual increase of cigarette consumption dropped to nearly zero, it soon sprang back to pre-campaign levels.^50^ Anti-smoking laws were sporadically enforced the following years, but it was not until the 2000s when coordinated efforts recommenced, often under the auspices of the European Community.

Nonetheless, policy efforts in the country have been historically futile.^51^ It is telling that several smoking bans have been legislated to date, but none have been practically enforced.^52^^,^^53^ Thus, the recent decline in smoking rates seems more closely related to the extreme income drop during the Great Recession rather than a tobacco control strategy *per se*. As shown in Fig. 4, real cigarette taxes did increase over the period. However, they remain below the European average,^54^ and it is questionable whether the government would have opted for a tax increase absent the need for public revenue. Thus, a relapse of the smoking epidemic once the economy recovers remains a possibility.

On a more theoretical level, our results contradict the prediction of the smoking epidemic model that smoking diffusion follows a hump-shaped pattern over time, which we do not find for Greek men. High persistence in smoking popularity among consecutive generations of men points to alternative explanations, e.g. the idea that smoking behavior is culturally determined.^55^ Indeed, the smoking epidemic model builds on US data and applies less accurately in countries with dissimilar culture (e.g. for women in China and the UK^27^). Further, because it relies on smoking popularity alone and ignores other dimensions of smoking (duration, frequency and quality), its accuracy is disputed even in the US context.^28^

### Limitations of this study

This latter limitation also applies here. The smoking trajectories presented are not temporally comparable in terms of exposure to health risk because they do not reflect the changing quantity and quality of cigarettes. The harmful content of cigarettes (e.g. tar) varies over time and cigarette consumption varies both across generations and over the life course. US research has shown that exploiting this variation improves the prediction of future smoking-related mortality.^28^ Unfortunately, time-series on cigarette content are not available for Greece. Also, the GATS does not ask ex-smokers to report the number of cigarettes they used to smoke. This information is available for current smokers only (see column 7 in Table 1). When we construct smoking trajectories standardized by the number of cigarettes for current smokers, a hump-shaped pattern emerges clearly (see online supplement).

We acknowledge that our analysis does not take into consideration second-hand smoking and consumption of smokeless tobacco (e.g. snuff or chewing tobacco) that were prevalent in the early sample period. Neither do we consider ‘vaping’ of e-cigarettes, which is the latest trend. This further obscures the intergenerational and temporal comparability of exposure to tobacco health risk. However, during the study period, first-hand smoking has been vastly widespread, overriding all the above. For example, <1% of the respondents report to have ever used smokeless tobacco daily or to smoke e-cigarettes daily during the survey year. Therefore, the historical narrative is sustained.

## Supplementary Material

Greek_smoking_epidemic_Appendix_fdab342

## Notes

**Rebekka Christopoulou,** Assistant Professor

**Georgios Mavropoulos,** PhD student

**Georgios Voucharas,** PhD student
