# Supplementary material for: The Greek smoking epidemic from a life-course perspective
Source: J Public Health (Oxf). 2021 Sep 8;44(4):e479–86. doi: 10.1093/pubmed/fdab342 (PMC9715303; doi:10.1093/pubmed/fdab342)
Supplement: Greek_smoking_epidemic_Appendix_fdab342 [file greek_smoking_epidemic_appendix_fdab342.docx]

**The Greek smoking epidemic from a life-course perspective**

**ONLINE SUPPLEMENT**

**Reconstruction of historical smoking prevalence**

The GATS (2013) survey asks questions on both contemporaneous and retrospective smoking behavior. The questions we use in our analysis are the following:

1. What is your tobacco usage status at present? (Every day, less than every day, none)

2. Have you used tobacco every day in the past?

3. What was your tobacco usage status in the past? (Every day, less than every day, none)

4. (If former or current smoker:) At what age did you first start to use tobacco every day?

5. (If former smoker:) How long ago did you quit using tobacco?

For more details, see: www.who.int/tobacco/surveillance/survey/gats/grc_country_report.pdf

To construct the life-course smoking trajectories we present in this article, we use retrospectively reported data on lifetime smoking status. As we explain in the main text, we assume that current and former smokers smoked in each year from the age they started until either the survey year (current smokers) or the age they said they quit (ex-smokers). Because we lack data on whether and when smokers might have temporarily quit, we assume that none of the smokers ever temporarily quit. Using these cross-sectional data, we then construct a longitudinal data set. In particular, for all respondents with valid responses, we construct a smoking status indicator in each year of life that equals 0 if a person does not smoke in that year and 1 if she or he does smoke. Finally, we group observations into categories by sex, and ten-year birth cohort. We measure smoking prevalence rates over the life-course as the mean smoking status in each year of the gender-specific cohort groups (weighted by sampling weights).

Because our calculations rely on retrospective smoking questions, we need to account for the fact that smokers are less likely than non-smokers to answer those questions because they are more likely to die from a smoking-related cause prior to the date of survey. Due to this differential mortality, we underestimate smoking rates for old cohorts. To correct for this bias, we apply the simple formula proposed by Harris(1) but use detailed cause-specific mortality data by age and gender, which we produce following the Peto et al.(2) technique. Previously published work explains this procedure and its advantages over alternative practices in detail (3, 4). That work uses data from the UK, US, Russia, and Spain to show that differential mortality significantly biases smoking rates reconstructed from retrospective reports only for cohorts who, at the time of the interview, were older than 60. Fully documented STATA codes, which can be used to correct for differential mortality bias, are freely available here: http://smoking-research.ehe.osu.edu/data-and-codes/.

Given that all this material is widely accessible, we do not fully describe the methods here. We do, however, list the exact data sources. For the period 1950-2013, we use population data and cause-specific deaths by age and gender from the World Health Organization (WHO) Mortality Database. For the period 1933-1949, we use total mortality rates by birth cohort and gender from the Human Mortality Database (HMD). For this period, i.e. when we have only overall mortality data, we assume that the relative mortality of smokers and non-smokers is time-invariant and equal to the mean relative mortality by cohort and gender derived from the cause-specific data. For years with no available mortality data, we simply back cast the differential mortality adjustment factor. As the reference population in our calculation of smoking-attributable mortality we use non-smokers from the Cancer Prevention Study II 1982-88 carried out by the American Cancer Society. This was provided by M.J. Thun, MD, MS; former Vice President Emeritus, Epidemiology, and Surveillance Research, American Cancer Society via electronic mail (February 2010). The CPS-II sample includes more than 1 million Americans above the age of 30 starting in 1982. Like other studies, we use the CPS-II because it provides mortality rates of some of the first cohorts of men to have smoked heavily and because it conveniently disaggregates them by sex and age.

In the figure S1 we present, for each cohort and gender, the smoking prevalence rates reconstructed from our retrospective data, unadjusted (solid line) and adjusted (dotted line) for smoking-related differential mortality bias. We only present results for cohorts over age 60 because, as we mentioned already, smoking related mortality differences for younger cohorts are small and the adjustment has negligible effects on smoking rates. We also use the Pearson test of independence for binary variables to test whether adjusted and unadjusted prevalence rates statistically differ. We find that the difference between unadjusted and adjusted smoking prevalence is zero for women but non-negligible for men. At the peak prevalence rate for men who at the year of survey were ages 80-89, 70-79 and 60-69 at the year of survey, adjusted and unadjusted prevalence rates differ by 6.2, 2.6, and 1 percentage points, respectively. However, because of small sample sizes (especially for the oldest cohort), the adjustment is never statistically significant.

**Figure S1. Adjustment of smoking prevalence for differential mortality bias by gender, birth-cohort and year.**

Finally, we note that, for clarity of exposition, all trajectories presented in the paper have been subjected to a standard least-squares smoothing. To ensure that there are no level-changes in the smoothed trajectories, we have used very conservative bandwidths in the smoothing procedure. For the trajectories of standardized and unstandardized smoking prevalence we used a bandwidth of 0.1, while for the trajectories of smoking-attributable mortality we used a somewhat higher bandwidth of 0.5 because the mortality data at hand are only available in 5-year age categories.

**Forward projections of smoking-attributable mortality**

To project the smoking-attributable mortality forward we rely on the estimates of Specification II augmented by some variables that capture more variation across and within cohorts, thus increasing precision (i.e. cohort fixed-effects and a variable that measures the average duration of smoking at each year of each cohort's life-time). As we explain in the main text, we only use the maximum values of the estimated smoking-mortality correlations. Of course, these estimates correspond to specific lag distances between smoking and mortality (19 years for men and 21 years for women). By basing mortality predictions on those estimates, we assume that at all other lag distances (conditional) smoking-mortality correlations are zero. Due to this assumption, we may overestimate future mortality for younger cohorts and underestimate it for older cohorts, a pattern which is evident in Figure S2. One can improve the accuracy of the projections by allowing for multiple lag distances in the model specification. Also, our forward projections require assumptions about the future evolution of the control variables. The results we present in Figure S2 are based on simple linear extrapolations of these variables, i.e. on the assumption that they will continue to change at the same constant rate.

**Figure S2. Observed versus predicted smoking-attributable mortality rates.**

Note: Lines from left to right correspond to cohorts born in 1944-1953, 1954-1963, 1964-1973, 1974-1983, 1984-1993, 1994-2003, respectively.

**Standardization of smoking rates by the number of smoked cigarettes (current smokers only)**

To standardize smoking rates by the number of smoked cigarettes, we redefine a smoker to be a person that smokes 20 cigarettes per day; i.e. we calculate: standardized smoking prevalence = unstandardized prevalence*(cigarettes/20). We present the standardized trajectories in Figure S3. Clearly, adjusting for cigarette consumption shifts the unstandardized smoking prevalence of each generation (presented in Figure 1 in the main text) either up or down, depending on whether that generation smoked more or less than a pack on average. As a result, standardized smoking patterns presented here form a hump-shaped pattern over time, as predicted by the smoking epidemic model.

**Figure S3.Life-course trajectories of smoking prevalence adjusted for the number of cigarettes smoked per day by gender and birth-cohort (current smokers only).**

**References**

1. Harris J. Cigarette-smoking among successive birth cohorts of men and women in the United-States during 1900-80. Jnci-Journal of the National Cancer Institute. 1983;71(3):473-9.

2. Peto R, Lopez A, Boreham J, Th un M, Heath C. Mortality from tobacco in developed-countries - Indirect estimation from national vital-statistics. Lancet. 1992;339(8804):1268-78.

3. Christopoulou R, Han J, Jaber A, Lillard D. Dying for a smoke: How much does differential mortality of smokers affect estimated life-course smoking prevalence? Preventive Medicine. 2011;52(1):66-70.

4. Lillard D, Christopoulou R, Lacruz A. RE: "Validation of a method for reconstructing historical rates of smoking prevalence". American Journal of Epidemiology. 2014;180(6):656-8.
